# Disturbed Mental Imagery of Affected Body-Parts in Patients with Hysterical Conversion Paraplegia Correlates with Pathological Limbic Activity

**DOI:** 10.3390/brainsci4020396

**Published:** 2014-05-20

**Authors:** Arnaud Saj, Noa Raz, Netta Levin, Tamir Ben-Hur, Shahar Arzy

**Affiliations:** 1 Department of Fundamental and Clinical Neurosciences, Faculty of Medicine, University of Geneva, Geneva 1211, Switzerland; E-Mail: arnaud.saj@unige.ch; 2 Department of Neurology, Hadassah Hebrew University Medical Center, Jerusalem 91120, Israel; E-Mails: noa.raz@mail.huji.ac.il (N.R.); netta@hadassah.org.il (N.L.); tamir@hadassah.org.il (T.B.-H.); 3 Neuropsychiatry Lab, Faculty of Medicine, Hadassah Hebrew University Medical School, Jerusalem 91120, Israel

**Keywords:** conversion disorder, hysteria, body-processing, mental-imagery, insular cortex, anterior cingulate cortex, fMRI

## Abstract

Patients with conversion disorder generally suffer from a severe neurological deficit which cannot be attributed to a structural neurological damage. In two patients with acute conversion paraplegia, investigation with functional magnetic resonance imaging (fMRI) showed that the insular cortex, a limbic-related cortex involved in body-representation and subjective emotional experience, was activated not only during attempt to move the paralytic body-parts, but also during mental imagery of their movements. In addition, mental rotation of affected body-parts was found to be disturbed, as compared to unaffected body parts or external objects. fMRI during mental rotation of the paralytic body-part showed an activation of another limbic related region, the anterior cingulate cortex. These data suggest that conversion paraplegia is associated with pathological activity in limbic structures involved in body representation and a deficit in mental processing of the affected body-parts.

## 1. Introduction

Conversion disorder (“hysteria”) is defined as neurological disturbances that cannot be attributed to neurological disease [1]. Although hysteria has been known for 2000 years and intensively investigated by 19th century neurologists, such as Charcot [2], Janet [3] and Freud [4], not much is known of its underlying functional mechanisms. While few neuropsychological findings have already demonstrated bifrontal impairment in conversion disorder [5], it has only recently become a subject for neuroimaging studies [6]. These studies showed altered patterns of perfusion in response to sensory stimulation of the affected area, especially in the contralateral prefrontal, parietal and subcortical regions [1,6,7,8,9,10,11,12]. Prefrontal cortex was found to be active during attempts to move the paralytic leg in chronic motor conversion, in comparison to the unaffected limb [13]. However, the dorsolateral region of the prefrontal cortex showed decreased activity, together with a network composed of inferior parietal cortex, cerebellum, contralateral primary sensorimotor and premotor cortex, suggesting inhibition of a network involved in programming and execution of movement by the prefrontal cortex [6,13,14,15,16]. While these studies suggest certain cortices to play a central role in conversion disorder, it is still not clear how the mental disturbance underlying conversion leads to the neurological deficit. Herein we investigated, using behavioral tasks and functional MRI, two young patients with acute conversion paraplegia related to stressogenic events. We hypothesized that paralysis was due to impairment in mental processing of the affected body-parts rather than motor execution.

## 2. Patients

Patient 1 was a 20-year-old highly-motivated right-handed soldier, with no neurological or psychiatric antecedents, appointed to an officer-training course. Several weeks before the course, he suddenly “collapsed” during physical training, with inability to walk. Detailed medical assessment did not reveal any deficit. The patient was discharged for rest at home and then returned to the army with no functional complaints. Shortly after, the paralysis recurred, though repeated medical workup was negative. His officer-training course was postponed for four months and the patient recovered again to a fully functional state. Two weeks before the newly-appointed time the patient collapsed again during physical effort. On admission he was fully conscious, complaining of inability to move both legs. Neurological examination revealed an inability to move the hips upon request, however, during maneuvers in bed and during an attempt to sit down there was unintentional use of hip muscles, and the Hoover test was positive. Neuropsychological examination and structural MRI were normal. His high motivation and recurrent attempts to become an officer ruled out malingering phenomena. In light of these findings the patient was diagnosed as suffering from relapsing conversion paraplegia in the probable context of conflict between his motivation to pass the officer course and his fear from failure.

Patient 2 was a 33-year-old single woman of north-African origin. The second out of seven siblings, her high academic achievement evoked interfamilial conflicts, particularly with her older brother. At work she suffered from repetitive struggles with her co-workers. The patient described that during these situations she developed “nervousness” that sometimes manifested as weakness in her lower limbs, which prevented her from working. These situations became increasingly frequent. The patient was brought to the emergency room after a major conflict in her work place, which was followed by complete paraplegia, saying that “my legs cannot carry my body anymore”. She was unable to move the hips upon request, however, during an attempt to sit down there was unintentional use of hip muscles, and the Hoover test was positive. Neuropsychological tests and structural MRI were normal. The patient was therefore diagnosed as suffering from relapsing conversion paraplegia in the probable context of professional, social and familial conflicts.

## 3. Methods

We used fMRI to test the activation pattern elicited during physical attempts to move and during mental imagery of moving the affected limb and the non-affected limb. Patients were presented with written names of body parts (legs/arms), which they were asked to slightly move. Then, a similar procedure was used, however patients were required to merely imagine moving their body-parts while no real movement or attempted movement was performed. The experiment was conducted using a block design paradigm of 18 experimental epochs, each lasting 12 s and followed by a 9 s rest period. Data analysis was performed using the Brain Voyager QX 1.8 software package (Brain Innovation, Maastricht, The Netherlands). Preprocessing included head motion correction, slice scan time correction, spatial smoothing (8 mm FWHM) and high-pass filtering (cut-off 180 s). A general linear model (GLM) was used to generate statistical parametric maps. Significance levels were FDR corrected (*p* < 0.05; for further details about fMRI acquisition and analyses see Supplementary Information).

We also compared patients’ performance in a mental-rotation task involving body-parts (legs and arms) and non-corporeal objects (letters) [17,18]. Patients and six age-matched healthy controls were presented with these stimuli oriented in five different angles (0°, 45°, 90°, 135°, 180°), which were either presented in a “normal view” or an “inverse view” (for body parts, the contralateral distal part was attached to the ipsilateral proximal part; alphanumeric characters were presented in a mirror-reversed view; see below). Patients and controls were requested to determine as quickly as possible whether the stimulus was presented normally or inversely. For patient 2, fMRI was conducted during task performance using a block design paradigm of 20 experimental epochs for each stimulus, in order to also search for a different activation pattern between affected and non-affected limbs (Supplementary Information). Patients and controls provided written informed consent, and the study was approved by the Ethics Committees of Geneva University and Hadassah Hebrew University Hospitals.

## 4. Results

**Figure 1:**
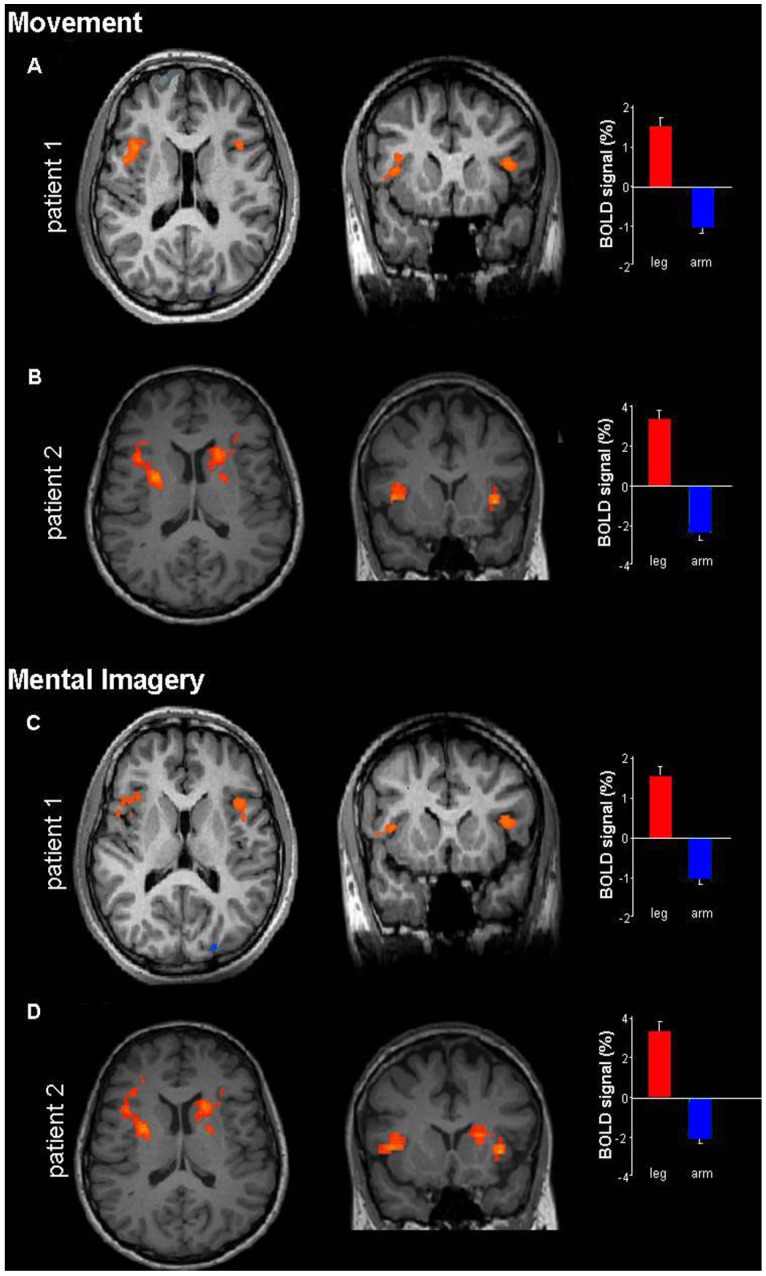
BOLD responses in the insular cortex during movement and mental-imagery conditions. Significant BOLD responses are superimposed on axial and coronal slices in the movement (**A**,**B**) and mental imagery (**C**,**D**) conditions for both patients. Percent signal change are shown on the right (leg: red; arm: blue). Note the similar patterns for the two patients as well as between movement and mental imagery conditions (error bars represent the standard error of estimate).

**Figure 2:**
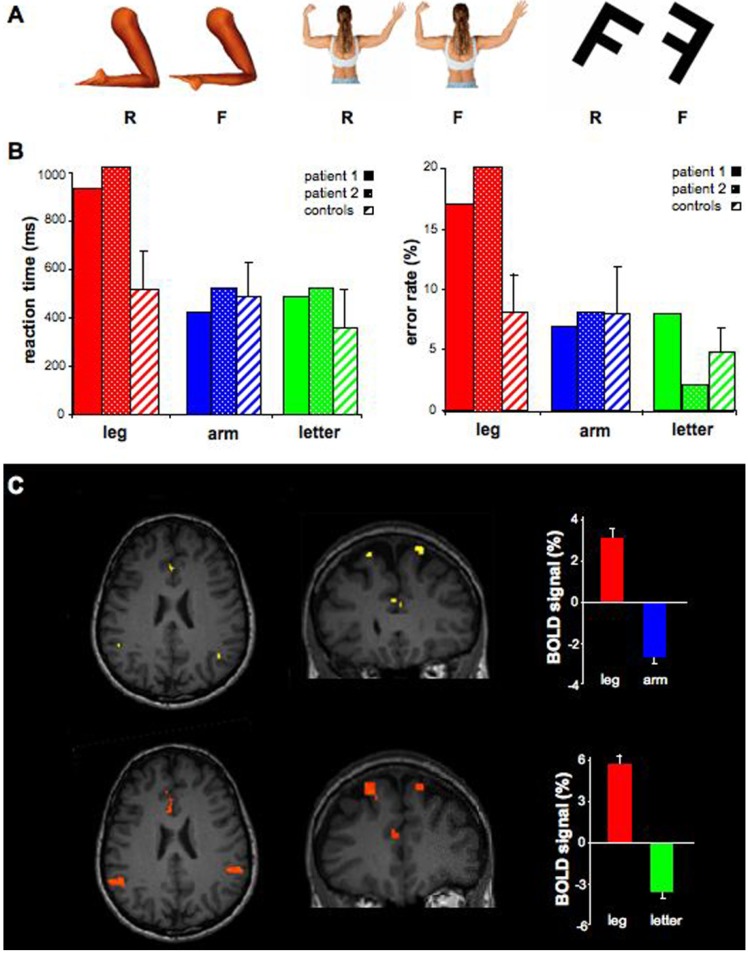
Mental rotation of legs, arms and letters. (**A**) Illustration of the stimuli used for tasks, including legs, arms and objects (letters), presented in right, normal view (R) or in false, inverse view (F). The stimuli were presented in five different angles (0°, 45°, 90°, 135°,180°). Patients and control subjects were requested to determine as quickly as possible whether the stimulus was the normal one or the inversed one. (**B**) Mean reaction-times (**left**) and error-rates (**right**) for legs (red), arms (blue) and letters (green) are plotted separately for patient 1 (plain bars), patient 2 (points) and control subjects (strips). Note the significantly higher reaction times for the legs in the patients with respect to control subjects. Error bars represent the standard deviation. (**C**) BOLD responses in the anterior cingulate and left parietal cortex during mental rotation of legs with respect to arms (**upper row**) and letters (**lower row**). Percent signal changes are shown on the right (red: leg; blue: arm; green: letter).

First, we compared fMRI activation upon attempt to move different body-parts, as well as during imagined movements. While comparing movements of the implicated body parts (legs) to non-implicated parts (arms), we found bilateral activation of the insular cortex, with right predominance (*p* < 0.01; Figure 1A,B). Remarkably, the same areas were also active during mental-imagery of moving the legs relative to the arms (*p* < 0.01; Figure 1C,D). The inverse contrast (arm > leg) showed an activity in the motor cortex. In order to further investigate the role of mental-imagery in conversion disorder, we made use of a task of mental rotation of the implicated body-parts (legs), non-implicated parts (arms) and letters (Figure 2A). Both patients had significantly longer reaction-times for rotation of the legs compared to the arms and letters (Figure 2B). Reaction-times for legs rotation were significantly longer in comparison to control subjects, unlike for arms or letters (Figure 2B). Similar patterns were found for the error-rates (Figure 2B). Statistical analyses (repeated measures ANOVAs and Scheffé *post-hoc* tests) for both patients showed significant differences between mental rotation of legs and arms (*p* < 0.05) and legs and letters (*p* < 0.05) but not between arms and letters (*p* > 0.8). Control subjects did not show a significant difference between legs and arms (*p* = 0.9), however, significant differences were found between legs and letters (*p* < 0.01) and arms and letters (*p* < 0.01). Finally, patients and controls showed a global mental rotation function for all stimuli (Figure S1). Patient 2 was further examined using the mental rotation task under fMRI. The areas that were activated during mental rotation of legs with respect to arms (*p* < 0.01) included the left inferior parietal and anterior cingualte cortex (ACC; Figure 2C). ACC activity was also found while legs were compared to letters, together with activity in the left inferior parietal cortex. Comparison of arms to legs or letters revealed activation in the inferior parietal cortex but not in the ACC. A gradual bilateral response was found in the insula for legs > arms > letters (right: 9, 3.5, −1.5 (% of BOLD signal); left: 1, −2.5, −4, respectively).

## 5. Discussion

In two young patients with conversion paraplegia the following observations were found: (1) activation of the insular cortex during an attempt to move the paralyzed limb, as well as during mental imagery; (2) significantly higher reaction-times and error-rates for mental-rotation of an image of the paralyzed limb than the non-paralyzed one or external object; (3) activation of the ACC during mental rotation of the paralyzed limbs. These observations are discussed regarding the role of mental-processing of one’s own-body in conversion paralysis, and regarding the functional role of the insula and the ACC in motor control and in sensorimotor and emotional coding of the human body. Disturbed mental-processing of body-parts is known in neurological deficits such as asomatognosia, agnosia, disembodiment of body-parts and multisensory disintegration [17], as might also be the case in conversion paralysis [18]. The current findings support the hypothesis that conversion paralysis may not simply reflect a deficit in the executive level (motor) but also affect cognitive processes already at the level of mental processing of the affected organ. This is also suggested by the behavioral results, demonstrating selective deficit in mental rotation of the affected organ. The fMRI data support this hypothesis by showing similar insular activation for both movement execution and mental-imagery, unlike normal subjects [19]. In addition, the fMRI data recorded during the mental-rotation task showed reserved activation of parietal cortex (normally recruited by this task), together with increased ACC activity that might relate to active inhibitory processes underlying conversion [20,21].

Our neuroimaging data showed that movement and mental imagery deficits in conversion paralysis is related to insular activation [15]. The insular cortex is considered a part of the limbic system that mediates emotional responses, and is connected to other limbic areas, such as the amygdala, temporal pole and entorhinal cortex, as well as prefrontal and orbitofrontal cortices [22,23]. In addition, it is directly connected to primary and secondary somatosensory and motor areas as well as to the supplementary motor area (SMA). In various abnormal mental states, such as schizophrenia, phobias or hallucinations, neuroimaging studies revealed an abnormal insular activation [23]. Therefore, it was hypothesized that the insular cortex might play a central role in conversion disorders [7,15,21,23]. Comparison of mental rotation of legs in comparison to arms or letters revealed activation of another limbic component—The ACC. This co-activation might relate to the conflict between instructions and intentions in conversion disorder [1,24] or to inhibitory process specifically related to the production of conversion disorder [20,21]. Limbic activation might bridge between the underlying neuropsychiatric trigger to the conversion-induced functional disability. This activation might also be related to our behavioral results showing deficit in mental rotation of affected body-parts, by inhibition of activation at posterior parietal cortices [17].

Motor and premotor cortex were not activated in the leg > arm comparison, corroborating previous studies [14,18,25,26]. Cojan and colleagues [27] used a go/no-go task in patients with unilateral paralysis. Motor preparation responses in the motor cortex remained intact, while preparing to move the paralytic limb elicited activation in ventro-medial prefrontal cortex and precuneus, which the authors hypothesized to inhibit motor activity. This is unlike the present study, in which patients with bilateral hysterical paralysis were asked to move the paralytic limb. This difference may represent two different mechanisms for motor control and self-monitoring in conversion. One mechanism, which involves more explicit inhibition, may relate to integrity of frontal (vmPFC) and parietal (precuneus) cortical networks [27]. The other may represent implicit inhibition (or disbelief [28,29]), relying on limbic-related structures (insula, ACC, amygdala) [21].

A major limitation of the current study is that it encompasses only two patients. However, it reflects a general difficulty in recruiting patients with hysterical paralysis, particularly with the same phenomenological features (bilateral paraplegia). Studies are therefore usually limited by small population size and heterogeneous patient populations [6]. Non-paralytic body-parts (arms) served as control to the paralytic body-parts (legs) in this study, rather than healthy controls.

## 6. Conclusion

Based on our clinical, behavioral, and neuroimaging evidence, we propose that the pathological form of conversion paraplegia might be found already in early phases of mental imagery and processing of the paralytic body-part. The insula and the ACC are proposed as key regions where emotional, conscious motivation, body-processing and sensorimotor activations interact, combining the psychological background and motor manifestations which underlie the functional basis of conversion disorder.

## Supplementary Files

Supplementary File 1 Supplementary Information (PDF, 229 KB)
