# Supplementary material for: Disturbed Mental Imagery of Affected Body-Parts in Patients with Hysterical Conversion Paraplegia Correlates with Pathological Limbic Activity
Source: Brain Sci. 2014 May 20;4(2):396–404. doi: 10.3390/brainsci4020396 (PMC4101484; doi:10.3390/brainsci4020396)
Supplement: Supplementary File 1 — Supplementary Information (PDF, 229 KB) [file brainsci-04-00396-s001.pdf]

Supplementary Information

Figure S1. Mental rotation function. Mean reaction times as a function of orientation are plotted separately for each patient and control subjects for legs (left), arms (middle) and letters (right).

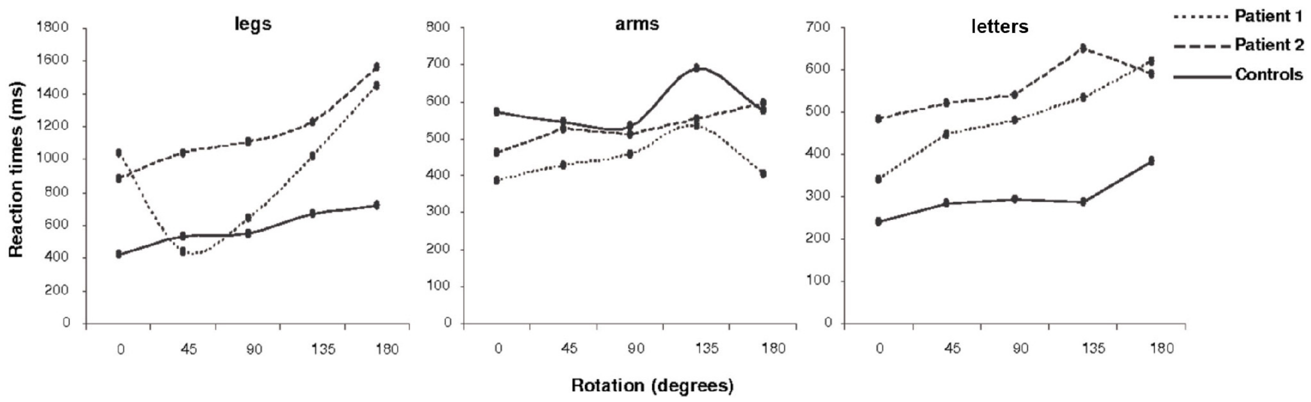

MRI Acquisition

The BOLD fMRI measurements were performed in a whole-body 3T Siemens scanner. The functional MRI protocols were based on a multi-slice gradient echo-planar imaging and a standard head coil. The functional data were obtained under the optimal timing parameters: T_R = 3 s, T_E = 50 ms, flip angle = 90°, imaging matrix = 64 × 64, FOV = 20 cm. The 32 slices with slice thickness 4 mm (with no gap) were oriented in the axial position. The scan covered the whole brain.

Stimuli and Experimental Paradigm

The present functional magnetic resonance imaging (fMRI) experiment tested the activation pattern elicited during real attempts to move or the mere imagery of moving different body parts. In the first experiment, patients were presented with written names of body parts (both legs, both hands and both ankles), which he was asked to move. A rest condition, in which the patients laid in the magnet with no action required, served as a hemodynamic baseline condition. The experiment was conducted using a block design paradigm of 18 experimental epochs, each lasting 12 s and followed by a 9 s rest period.

In a second experiment the identical procedure was used, however here patients required to mere imaging moving their body parts while no real movement or attempt to movement was performed.

Data Analysis

Imaging Data

Data analysis was performed using the Brain Voyager QX 1.8 software package (Brain Innovation, Maastricht, The Netherlands). In order to remove drifts and to improve the signal to noise ratio, functional images were realigned, corrected for slice timing, normalized to an EPI-template (re-sampled at a voxel-size of 3 mm), spatially smoothed (8 mm FWHM), and high-pass filtered (cutoff 180 s). A general linear model (GLM) was used to generate statistical parametric maps (modeling the hemodynamic response function using parameters as in Boynton *et al.*, 1996 [1]). This was done after the voxel activation time courses were transformed into Talairach space (Talairach and

Tournoux, 1988 [2]), *Z*-normalized and concatenated. Significance levels were calculated using the less stringent false discovery rate (FDR) correction term (Genovese *et al.*, 2002 [3]). The minimum significance level, corrected for any given cluster was $p < 0.05$. The averaged signal change during stimuli presentation (shifted by 1 T_R) was also calculated.

References

1. Boynton, G.M.; Engel, S.A.; Glover, G.H.; Heeger, D.J. Linear systems analysis of functional magnetic resonance imaging in human V1. *J. Neurosci.* **1996**, *16*, 4207–4221.
2. Talairach, J.T.P.; Tournoux, P. *Co-planar Stereotaxic Atlas of the Human Brain*; Thieme Medical Publisher: New York, NY, USA, 1988.
3. Genovese, C.R.; Lazar, N.A.; Nicols, T. Thresholding of statistical maps in functional neuroimaging using the false discovery rate. *Neuroimage* **2002**, *15*, 870–878.

© 2014 by the authors; licensee MDPI, Basel, Switzerland. This article is an open access article distributed under the terms and conditions of the Creative Commons Attribution license (http://creativecommons.org/licenses/by/3.0/).
